# Metabolic Profiling Reveals Organ‐Specific Molecular Pathologies and Aging‐Associated Biomarkers in Progeroid Laminopathy

**DOI:** 10.1002/smmd.70042

**Published:** 2026-07-11

**Authors:** Lidan Hu, Xiaoyang Zhao, Yingxian Zhang, Jingjing Wang, Guozhen Wang, Xiaoli Liu, Zhan Feng, Jiazhen Yin, Zhaoyang Peng, Langping Gao, Jianhua Mao, Ning Shen

**Affiliations:** ^1^ Department of Nephrology, Children's Hospital, National Clinical Research Center for Children and Adolescents' Health and Diseases Zhejiang University School of Medicine Hangzhou Zhejiang China; ^2^ School of Basic Medical Sciences and Forensic Medicine Hangzhou Medical College Hangzhou Zhejiang China; ^3^ Liangzhu Laboratory Zhejiang University School of Medicine Hangzhou Zhejiang China; ^4^ Zhejiang Hospital Affiliated to Zhejiang University School of Medicine Hangzhou Zhejiang China; ^5^ Hangzhou Hospital of Traditional Chinese Medicine Hangzhou Zhejiang China

**Keywords:** aging, biomarker discovery, Hutchinson–Gilford progeria syndrome, multi‐omics, progeroid laminopathy

## Abstract

The molecular mechanisms linking premature and physiological aging remain incompletely understood. Here, we present a cross‐species, multi‐organ metabolic atlas of progeroid laminopathy (PL) generated through integrated lipidomic and metabolomic profiling of heart, lung, skin, and serum from Hutchinson–Gilford progeria syndrome (HGPS) mouse models and human PL cohorts. We identify organ‐specific metabolic alterations, with the most pronounced changes observed in the skin at the omics level. Notably, we uncover disruption of a taurine–TCA–TUDCA cytoprotective axis, suggesting a coordinated metabolic vulnerability in PL. Serum profiling further reveals candidate circulating biomarkers, including increased LPC(18:1e) and LysoPA along with decreased D‐glucose 1‐phosphate and NAD. These alterations are conserved across species and, importantly, display concordant trends in independent cohorts of physiologically aged healthy individuals. This overlap indicates a partial convergence between PL‐associated and normal aging‐related metabolic changes. Together, our findings provide a metabolic framework for understanding PL pathophysiology and highlight clinically accessible biomarkers that warrant future longitudinal validation.

## Highlights

- Organ‐Specific Resolution: We demonstrate that metabolic dysregulation in PL is highly tissue‐dependent, with the skin showing the most profound lipidomic remodeling among the analyzed tissues (specifically ceramide accumulation) and the heart exhibiting distinct glycolytic shifts.
- A Candidate Metabolic Axis: We observe a systemic disruption in the taurine‐TCA‐TUDCA metabolic axis across multiple organs in HGPS mice. This finding raises the hypothesis that impaired cytoprotective bile acid metabolism may contribute to multi‐organ vulnerability in PL.
- Translational Biomarkers: By intersecting mouse and human datasets, we identified and validated a panel of circulating biomarkers—specifically LPC(18:1e), LysoPA, and D‐glucose 1‐phosphate. Crucially, we demonstrate that these markers are associated with PL and show overlapping alterations with physiological aging in a healthy elderly cohort.

## Graphical abstract

This study integrates transcriptomic, lipidomic, and metabolomic profiling of the heart, lung, skin, and serum from Hutchinson–Gilford progeria syndrome (HGPS) mouse models and human progeroid laminopathy (PL) cohorts. Through multi‐organ analyses, we reveal organ‐specific aging signatures, and importantly, cross‐species serum metabolomics identify conserved circulating biomarkers—including elevated lysophosphatidylcholine (LPC) and lysophosphatidic acid (LysoPA), and reduced D‐glucose 1‐phosphate—that overlap with metabolic changes in physiological aging. Furthermore, targeted validation in independent PL and elderly cohorts confirms their potential as accessible biomarkers linking pathological and physiological aging.

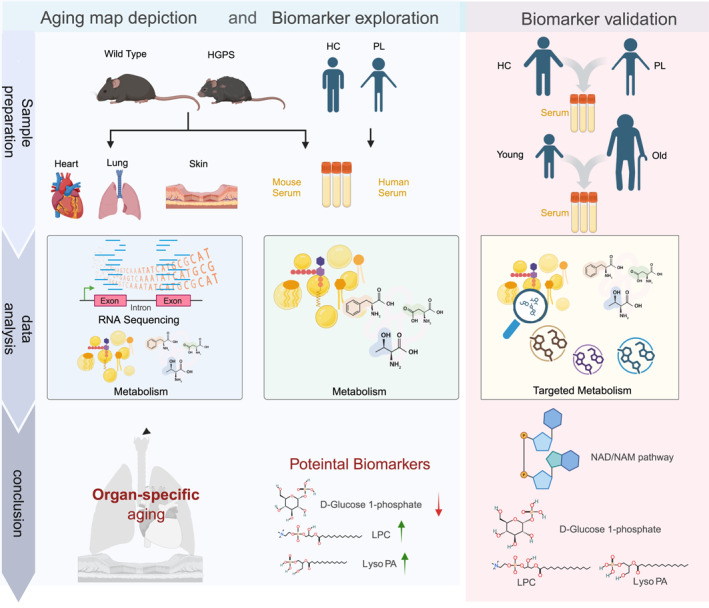

## 1 Introduction

Aging is a progressive biological process marked by declining physiological integrity and increased susceptibility to chronic diseases [1]. At the cellular level, it is driven by conserved hallmarks such as genomic instability, telomere shortening, mitochondrial dysfunction, and chronic inflammation. Due to the long timescale and heterogeneity of human aging, progeroid syndromes have emerged as valuable models for studying accelerated aging [2]. Among these, progeroid laminopathies (PLs), caused by mutations in the *LMNA* gene, shares key molecular and phenotypic features with physiological aging [3]. The most severe form, Hutchinson–Gilford progeria syndrome (HGPS) arises from a de novo mutation (c.1824C>T, G608G) and is characterized by systemic premature aging phenotypes [4].

To investigate PL pathophysiology, we employed an integrated multi‐omics approach with a focus on metabolomics and lipidomics, which provide a functional readout of cellular physiology [5]. Given that hallmark PL features—such as lipodystrophy and bioenergetic impairment—are inherently metabolic [6], this strategy enables more direct characterization of disease processes. In addition, circulating metabolites are readily accessible in clinical settings, enabling the identification of non‐invasive biomarkers with potential translational value.

Although prior studies have revealed molecular alterations in PL [7], comprehensive multi‐organ investigations that integrate metabolic data remain limited. To address this knowledge gap, we selected three representative organs—the heart, lung, and skin—for integrated molecular analysis. The heart and skin are well‐established sites of pathology in HGPS and display overt structural and functional decline [8]. The lung remains understudied. Its high metabolic activity and large surface area render it vulnerable to oxidative stress and environmental damage. Our prior work confirms premature aging phenotypes in lung tissue from both HGPS patients and mouse models, supporting its role in PL pathophysiology [9].

In this study, we employed lipidomics, and metabolomics to systematically characterize the molecular alterations across heart, lung, skin, and serum in a well‐established HGPS mouse model. To facilitate clinical translation and enable non‐invasive monitoring, we next focused on identifying accessible, circulating biomarkers. Specifically, we conducted untargeted metabolomic profiling in a cohort of PL patients. To the best of our knowledge, our cohort represents the largest known dataset of serum‐based metabolomic profiles in PL to date. Our study establishes a cross‐species, organ‐resolved metabolic atlas of PL and nominates circulating metabolites, such as LPC (18:1e), LysoPA (16_0_0_0), and D‐glucose 1‐phosphate, as candidate circulating metabolites associated with PL. Additionally, through an independent cohort of healthy elderly individuals, we revealed the potential of both LPC and LysoPA as metabolic features associated with physiological aging. Our data and findings may have important implications for the understanding of PL and aging.

## 2 Results and Discussion

### 2.1 Study Design and Phenotypic Characterization of HGPS Mice

**FIGURE 1:**
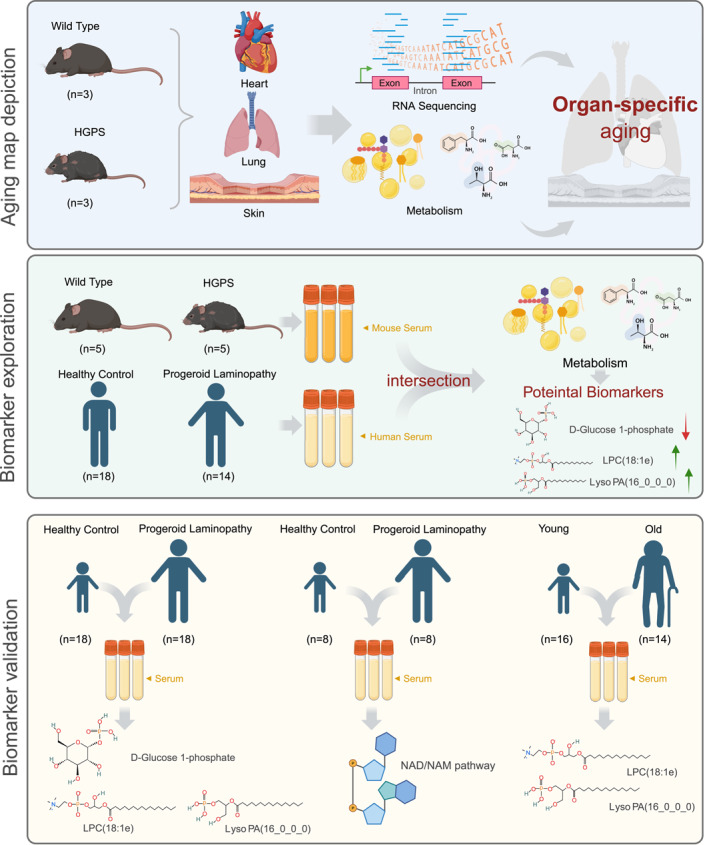
Technical roadmap of the experiment. Top panel (Aging map depiction): Heart, lung, and skin tissues were collected from WT and HGPS mice (*n* = 3 per group) for metabolomic profiling to map organ‐specific aging signatures. Middle panel (biomarker exploration): Serum samples from WT and HGPS mice (*n* = 5, each), and from the PL cohort (PL patients: *n* = 14, HC: *n* = 18), were analyzed by metabolomics to identify conserved circulating biomarkers. Bottom panel (Biomarker validation): Candidate biomarkers—including D‐glucose 1‐phosphate, LPC(18:1e), LysoPA(16_0_0_0), and metabolites in the NAD/NAM pathway—were validated in an independent PL cohort (PL patients: *n* = 18, HC: *n* = 18) and a physiological aging cohort (young people: *n* = 16, old people: *n* = 14).

To elucidate the systemic aging landscape in PL, we established a multi‐tiered experimental framework involving both HGPS mouse models and human cohorts, in which metabolomic and lipidomic profiling was applied to dissect tissue‐specific and circulating molecular alterations (Figure 1). First, wild type (WT) and homozygous HGPS (*Lmna* ^*G608G/G608G*^) mice were used to collect heart, lung, and skin tissues for histological characterization, and metabolomics analysis, aiming to map tissue‐specific metabolic alterations associated with premature aging. Second, to explore circulating biomarkers reflective of aging phenotypes, we conducted untargeted metabolomics profiling of serum samples from both mouse models and human cohorts (including age‐matched healthy control (HC) and PL patients). Third, the candidate biomarkers identified in the discovery analysis were validated in an independent cohort of PL patients and age‐matched healthy individuals through targeted metabolite quantification. Lastly, based on biomarkers validated in PL patients, we further examined their application potential in physiological aging in an additional aging cohort.

To investigate tissue‐specific molecular alterations in HGPS, we employed homozygous HGPS mice established and characterized in a prior study [9]. Compared to WT, HGPS mice exhibited markedly reduced body size and weight (Supporting Information S1: Figure S1A–C). Histological analysis revealed hallmark pathological features of the heart, lung, and skin in HGPS (Supporting Information S1: Figure S1D). These organ‐level abnormalities provide a foundation for subsequent metabolic analyses.

### 2.2 Metabolomic Profiling Reveals Lipid and Metabolic Dysregulation in HGPS Mice

Based on the pronounced organ‐level pathological alterations observed in HGPS mice, we next investigated whether these changes were accompanied by coordinated alterations in tissue metabolism. To investigate lipid composition alterations in PL, we performed mass spectrometry–based lipidomic profiling to analyze lipid profiles in the heart, lung, and skin of HGPS and WT mice. Orthogonal Partial Least Squares Discriminant Analysis (OPLS‐DA) revealed clear separation between HGPS and WT samples across organs with high intra‐group consistency (Supporting Information S1: Figure S2A), indicating significant lipidomic differences between genotypes.

**FIGURE 2:**
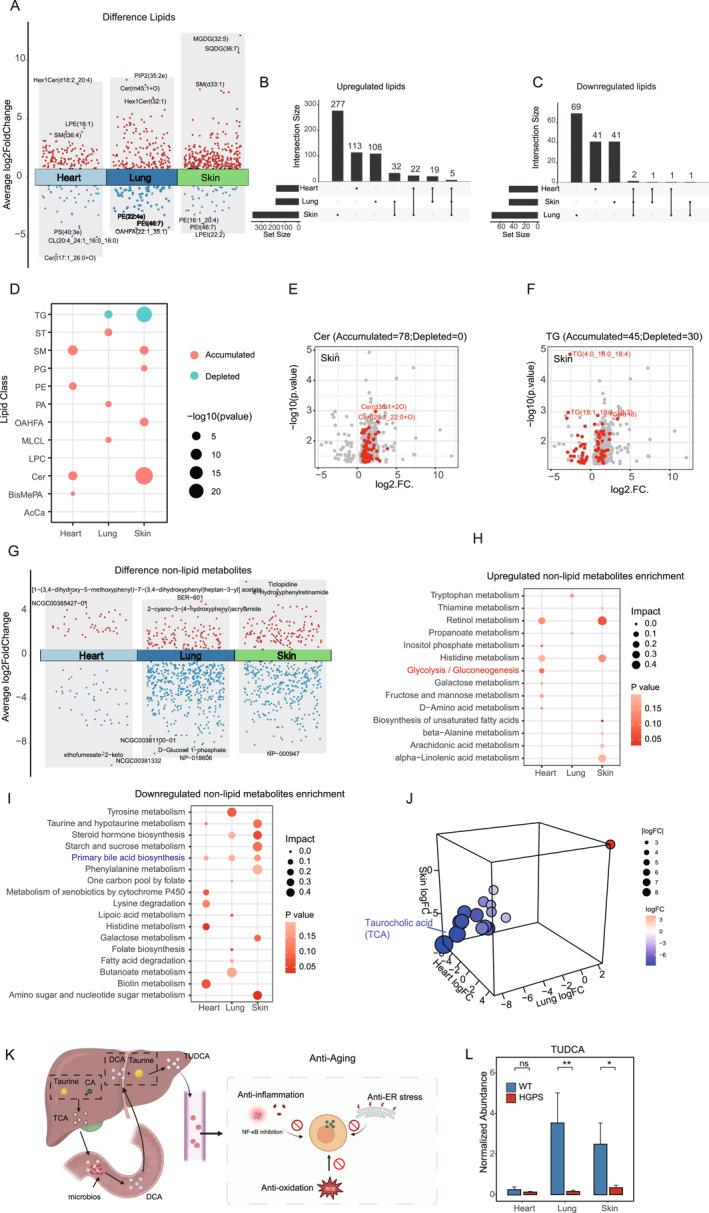
Metabolomic analysis of different organs in HGPS mice. (A) Volcano plot of differential lipids (HGPS vs. WT) across different organs, with red indicating significantly upregulated lipids and blue indicating significantly downregulated lipids. (B) Upset plot showing the number of significantly upregulated lipids unique to or shared across different organs. (C) Upset plot showing the number of significantly downregulated lipids unique to or shared across different organs. (D) Enrichment analysis of differential lipid species in different organs. (E) Volcano plot of differential lipids in the skin (HGPS vs. WT), with red indicating differential lipids belonging to the Cer class and gray indicating those not belonging to the Cer class. (F) Volcano plot of differential lipids in the skin (HGPS vs. WT), with red indicating differential lipids belonging to the TG class and gray indicating those not belonging to the TG class. (G) Volcano plot of differential hydrophilic metabolites in different organs (HGPS vs. WT). Red indicates significantly upregulated metabolites, and blue indicates significantly downregulated metabolites. (H) Enrichment of metabolic pathways for significantly upregulated metabolites across different organs. (I) Enrichment of metabolic pathways for significantly downregulated metabolites across different organs. (J) 3D scatter plot showing the expression changes of differential metabolites shared across three organs. (K) Schematic diagram of TCA‐to‐TUDCA conversion and anti‐aging mechanisms. In the liver, taurine conjugates with cholic acid (CA) to form TCA, which is then secreted into the intestine. Within the gut, TCA interacts with the gut microbiota and is partially transformed into deoxycholic acid (DCA). DCA re‐enters the liver through the enterohepatic circulation where it can be reconjugated with taurine to generate TUDCA. TUDCA is subsequently released into the bloodstream where it exerts anti‐aging effects. (L) Bar plot showing the normalized abundance of TUDCA in heart, lung, and skin tissues from WT (blue) and HGPS (red) mice.

Comparative analysis showed a higher number of upregulated than downregulated lipids across all three organs (Figure 2A–C) (Table S1). Notably, the skin exhibited the largest number of upregulated lipids among the three analyzed organs, whereas the lung exhibited the most downregulated lipids (Figure 2B,C), suggesting that lipid metabolism in these tissues may be particularly vulnerable to PL‐associated changes, as modeled in HGPS.

Lipid saturation status is a key determinant of membrane fluidity and signaling, with unsaturated fatty acids playing a particularly important role in aging‐related processes [10]. We next examined unsaturated lipid profiles and found that unsaturated lipid levels in the skin were markedly reduced in HGPS mice compared to WT controls (Supporting Information S1: Figure S2B), consistent with previous reports that changes in unsaturated lipid levels may contribute to aging‐associated pathologies [11, 12].

To further explore lipid class‐specific alterations, differential lipids were categorized into 12 distinct lipid classes (Figure 2D). Among them, Bis(methylthio)phosphoryl acetic acid (BisMePA)—a bisphosphonate lipid previously reported to accumulate in aging mouse hearts—was significantly elevated in the hearts of HGPS mice (Figure 2D, Supporting Information S1: Figure S2C) [13]. This finding highlights a shared lipid remodeling feature between HGPS and physiological aging, reinforcing the notion that HGPS recapitulates key aspects of natural aging at the molecular level.

In the skin, ceramides (Cer) were markedly enriched, with all differentially expressed Cers being upregulated in HGPS (Figure 2E). Cers are critical for maintaining skin barrier integrity, and their dysregulation is associated with various dermatological disorders [14, 15]. Thus, abnormal Cer accumulation may contribute to the cutaneous manifestations observed in HGPS, reflecting PL‐specific skin pathology. Conversely, triglyceride (TG)‐associated lipid levels were significantly reduced in both the skin and lung (Figure 2D). Although TG‐related lipids appeared among both upregulated and downregulated species, they constituted over 50% of the downregulated lipids but less than 25% of the upregulated lipids in the skin (Figure 2F, Supporting Information S1: Figure S2D). Given the essential role of TGs in providing lubrication and hydration for the skin barrier [16], TG depletion may be associated with compromised skin resilience against external stressors.

Regarding metabolomic analysis, the number of significantly upregulated species was lower than that of downregulated ones across all organs (Figure 2G) (Table S1). Among these, the skin exhibited the highest number of upregulated metabolites, while the lung had the highest number of downregulated metabolites. The heart showed the fewest alterations in both upregulated and downregulated metabolites (Supporting Information S1: Figure S2E,F). These results suggest that HGPS has a more pronounced impact on metabolic profiles in the skin and lung at the molecular level, while its effect on the heart is relatively limited.

Pathway enrichment analysis revealed organ‐specific metabolic alterations (Table S1) (Figure 2H,I). Glycolysis/gluconeogenesis pathways were exclusively enriched in the heart, consistent with metabolic reprogramming associated with cardiac aging [17]. This may reflect mitochondrial dysfunction and increased oxidative stress.

Cross‐organ analysis identified 1 commonly upregulated and 14 commonly downregulated metabolites among heart, lung, and skin tissue (Figure 2J). Among these, taurocholic acid (TCA) was notably downregulated across all three organs (Figure 2J). TCA is known to be converted in the body into tauroursodeoxycholic acid (TUDCA), a bile acid derivative with demonstrated antioxidative, anti‐inflammatory, and cytoprotective properties [18, 19, 20], which also plays an important role in anti‐aging processes (Figure 2K) [21, 22]. We found that TUDCA was significantly downregulated in the lungs and skin of HGPS mice, with a similar downward trend observed in the heart, although not statistically significant (Figure 2L). Given the potential of TCA to be metabolically converted into TUDCA and exert downstream protective effects, supplementation of TCA—or targeting the broader taurine‐associated bile acid metabolism—might represent a promising therapeutic strategy to mitigate disease progression in progeroid laminopathies.

### 2.3 Serum Metabolome Profiling Reveals Systemic Metabolic Dysregulation in PL Patients

After establishing organ‐specific metabolic alterations in HGPS mice, we then sought to identify aging‐related metabolic biomarkers in the serum of both HGPS mice and PL patients. The clinically accessible serum metabolites may serve as ideal biomarkers reflective of systematic aging.

**FIGURE 3:**
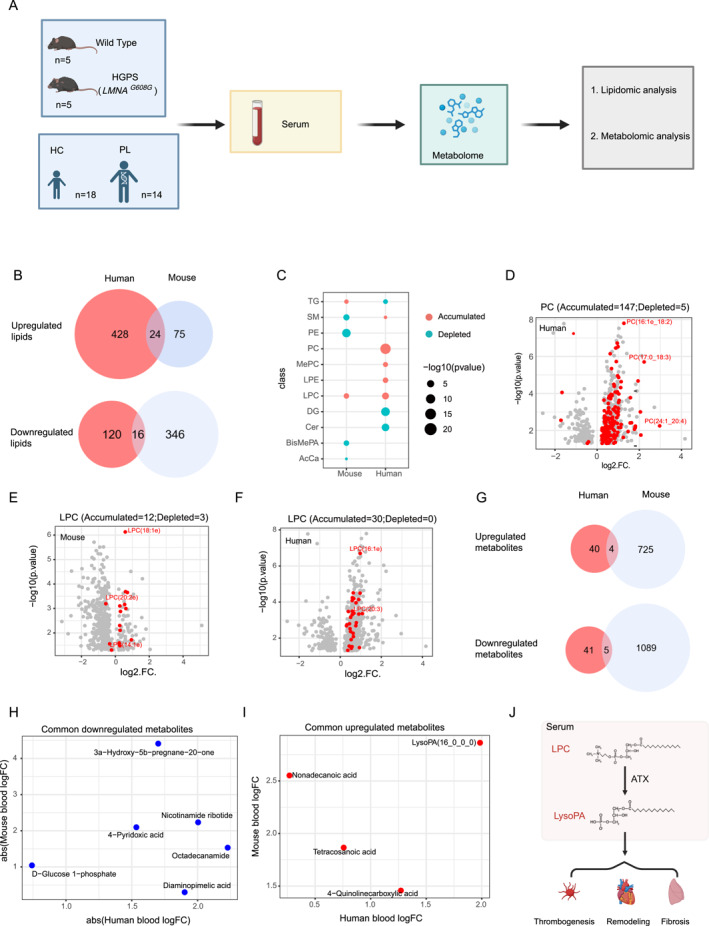
Serum metabolome analysis. (A) Schematic diagram of the experimental design. (B) Overlap of upregulated and downregulated lipids in serum samples from PL patients and mice. (C) Enrichment analysis of differential lipid classes in human and mouse serum. (D) Volcano plot of differential lipids in human serum (PL vs. WT), with red indicating lipids belonging to the PC class, and gray indicating lipids not belonging to the PC class. (E) Volcano plot of differential lipids in mouse model serum (HGPS vs. WT), with red indicating lipids belonging to the LPC class, and gray indicating lipids not belonging to the LPC class. (F) Volcano plot of differential lipids in human serum (PL vs. WT), with red indicating lipids belonging to the LPC class, and gray indicating lipids not belonging to the LPC class. (G) Overlap of upregulated and downregulated metabolites in serum samples from PL patients and HGPS mice. (H) Metabolites that are commonly downregulated in serum samples from the PL patients and HGPS mice, along with the fold change in serum levels in both species. *X*‐axis: Absolute value of the Log2‐transformed fold change of differential metabolites in the serum of HGPS patients compared to normal individuals. *Y*‐axis: Absolute value of the Log2‐transformed fold change of differential metabolites in the serum of HGPS mouse models compared to WT. (I) Common upregulated lipids in serum samples from PL patients and HGPS mice, and the degree of change in these lipids in both human and mouse serum. The *X*‐axis represents the fold change of differential lipids in the serum of PL patients compared to normal controls, while the *Y*‐axis represents the fold change of differential lipids in the serum of HGPS mice compared to WT mice. (J) Mechanism of LPC conversion to LysoPA in serum and its pathological effects on target organs.

To investigate alterations in circulating metabolites in PL, we performed lipidomic and metabolomic analyses on serum samples collected from HC, PL patients (including classical HGPS and HGPS‐like PL), WT mice and HGPS mice (Figure 3A). OPLS‐DA revealed tight clustering within species (human or mouse), indicating good biological reproducibility, while clear separation was observed between PL (including HGPS) and HC groups (Supporting Information S1: Figure S3A), suggesting substantial differences in circulating lipid compositions.

Differential analysis showed that a total of 452 lipids were upregulated and 136 lipids were downregulated in the serum of PL patients, while 99 lipids were upregulated and 362 lipids were downregulated in HGPS mice (Figure 3B) (Table S2). Among these, 24 lipids were commonly upregulated in both PL patients and HGPS mice. For downregulated lipids, 136 lipids were significantly reduced in the serum of PL patients, while 362 lipids were significantly downregulated in HGPS mice, with 16 lipids being commonly downregulated in both species (Figure 3B).

To further explore lipid class‐specific alterations, we first categorized the differential lipids into 12 major lipid classes (Figure 3C). Further classification revealed that upregulated lipids were predominantly enriched in LPCs and PCs, while downregulated lipids were mainly diacylglycerols (DGs) and Cers (Supporting Information S1: Figure S3B,C). In the serum of PL patients, PC levels were significantly elevated, with 147 out of 152 differentially expressed PC species being upregulated and only 5 downregulated (Figure 3D), suggesting a strong association between elevated PC levels and PL pathology. The alterations in LPCs were also remarkable. In HGPS mice, 12 of the 17 differentially expressed LPCS were upregulated (Figure 3E), while all 30 differentially expressed LPCS were upregulated in PL patients (Figure 3F), with LPC(18:1e) being the most significantly changed. Further analysis showed that shorter‐chain LPCs (such as the previously mentioned LPC 18:1e and LPC 18:2e, which were commonly upregulated in both PL patients and HGPS mice) exhibited greater changes (Supporting Information S1: Figure S3D,E). These findings indicate that shorter‐chain LPCs may be more sensitive to PL progression, particularly in the context of HGPS, and further support the potential of LPCs as conserved metabolic features associated with PL.

In the metabolite analysis, OPLS‐DA again demonstrated good intra‐group reproducibility and clear intergroup separation (Supporting Information S1: Figure S3A). Differential analysis revealed 44 upregulated and 46 downregulated metabolites in human PL serum, and 729 upregulated and 1094 downregulated species in mouse PL serum (Figure 3G) (Table S2). Among these, 4 metabolites were commonly upregulated and 5 were commonly downregulated between species (Figure 3G).

Among commonly downregulated metabolites (Figure 3H), D‐Glucose 1‐phosphate was significantly reduced in the serum of PL patients and HGPS mice. D‐Glucose 1‐phosphate is a key intermediate in glycogen synthesis and glycogenolysis (Supporting Information S1: Figure S3F). Consistent with this, qPCR analysis revealed that genes encoding the enzymes involved in its synthesis were downregulated in HGPS mouse tissues, whereas genes encoding enzymes promoting their breakdown were upregulated (Supporting Information S1: Figure S3G,H). These results indicate an imbalance in glucose metabolism in PL, a process known to accelerate aging through increased glycation and oxidative stress [23].

Among commonly upregulated metabolites, LysoPA (16_0_0_0) exhibited the greatest increase in both species (Figure 3I). Previous studies revealed that LPC can be converted into LysoPA in the serum via enzyme autotaxin (ATX) (Figure 3J). In our previous analysis of lipid metabolites in the serum, we found that LPC levels were significantly elevated in both PL patients and mice (Figure 3E, F). Given the significant elevation of LPC observed in our serum data, it is plausible to speculate that increased LysoPA levels may be attributable to the increased availability of LPC substrates potentially processed by ATX, although the direct enzymatic activity of ATX remains to be further investigated in our specific cohorts. Functionally, LysoPA has been implicated in multiple organ pathologies: promoting pulmonary fibrosis via LPA1 [24, 25], mediating cardiac remodeling through LPA3 [26, 27], and promoting thrombosis by enhancing fibronectin‐matrix interactions on platelets [28]. Therefore, elevated LPC and LysoPA levels may contribute to systemic organ dysfunction in PL patients (Figure 3J).

### 2.4 Validation of Candidate Biomarkers in PL Patients and Aging Cohorts

**FIGURE 4:**
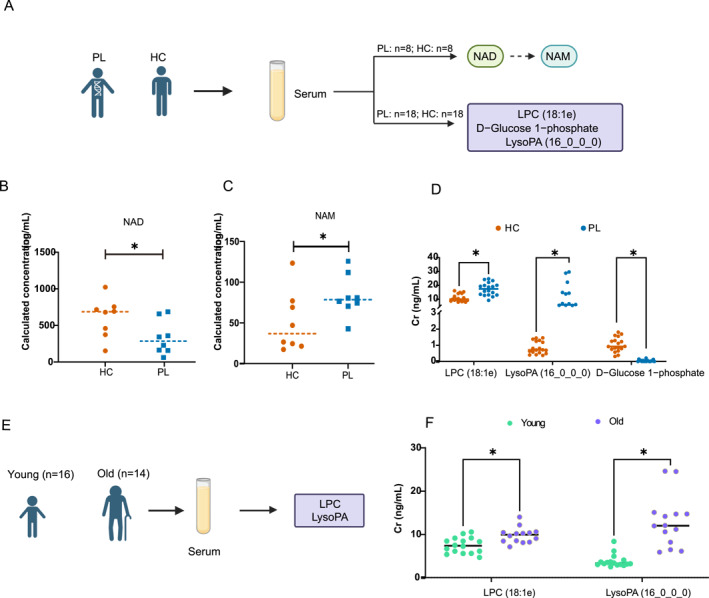
Biomarker assessment in PL patients. (A) Schematic overview of human serum sample collection and analysis. For PL validation, serum from HC (*n* = 18) and PL patients (*n* = 18) was used to quantify NAD, NAM (*n* = 8), and other circulating metabolites, including LPC, LysoPA, and D‐glucose 1‐phosphate (*n* = 18). (B) Quantification of serum NAD levels in HC and HGPS groups. (C) Quantification of serum NAM levels in HC and PL groups. (D) Quantification of LPC, LysoPA, and D‐glucose 1‐phosphate levels in HC and PL serum. (E) Schematic overview of human serum sample collection and analysis. For aging validation, serum from young (*n* = 16) and old individuals (*n* = 14) was collected to assess LPC and LysoPA levels. (F) Quantification of LPC and LysoPA levels in young and old individuals. ns: not significant. *: *p* < 0.05.

To validate candidate biomarkers identified from our metabolomic and lipidomic screening, we analyzed serum samples from 18 PL patients and age‐matched HC (Figure 4A). The ratio of male is 66.6% in both the PL and HC groups. Mean age was 49.83 months (95% CI: 24.58–75.08 months) in the PL group and 72.06 months (95% CI: 46.17–97.94 months) in the HC group. There were no significant differences in age or sex between the two groups (*p* = 0.2035). We first investigated the perturbation of the NAD metabolism pathway in these patients and observed a significant reduction in NAD levels in PL patients (Figure 4B), accompanied by pronounced accumulation of downstream metabolites of NAD, such as NAM, 2‐ pyridine (PY) and 4‐PY (Figure 4C, Supporting Information S1: Figure S4A,B). Concordantly, The NAD/NADH ratio, previously reported to decline with aging [29], was significantly reduced in the PL group (Supporting Information S1: Figure S4C,D). These NAD metabolic signatures closely resemble patterns observed during physiological aging. Moreover, LPC and LysoPA levels were significantly elevated, while D‐Glucose 1‐phosphate levels were markedly decreased in PL patients compared with controls (Figure 4D), consistent with our earlier metabolomic findings.

To explore the broader applicability of these biomarkers to physiological aging, we further tested them in a separate cohort comprising 16 HC (mean age ∼47.76 months; 95% CI: 33.00–62.52; 37.5% male) and 14 elderly individuals aged over 65 years (mean age ∼73.5 years; 95% CI: 70.68–76.32; 37.5% male) (Figure 4E). Notably, LPC and LysoPA levels were significantly elevated in elderly individuals compared with children (Figure 4F), suggesting that LPC and LysoPA may serve as candidate aging‐associated biomarkers in PL and aging.

## 3 Discussion

Progeroid laminopathies (PLs) are a systemic disease that profoundly affects metabolic processes across multiple organs [30], yet comprehensive multi‐organ characterizations remain scarce. Here, we provide a systematic metabolic characterization integrating lipidomic and metabolomic analyses from heart, lung, and skin tissues of HGPS mouse models, complemented by serum profiling from both mice and human cohorts. This framework uncovers organ‐specific aging‐like signatures and identifies circulating biomarkers that overlap between pathological and physiological aging.

Notably, although cardiovascular events are the primary cause of death in PL patients [31], the heart exhibited the fewest metabolic changes. This paradox may reflect the heart's high sensitivity to molecular disruption, where minimal alterations could trigger severe outcomes such as heart failure or arrhythmia [32, 33]. Pathway analysis revealed significant enrichment of energy metabolism dysregulation in the heart, suggesting that impaired bioenergetics may underlie cardiac vulnerability in PL. In contrast, the skin displayed the most extensive molecular dysregulation based on our multi‐omics analyses. Lysosomal dysfunction—a known driver of aging—was prominent, suggesting a potential contribution to accelerated aging processes through impaired autophagy and accumulation of damaged cellular components [34]. Downregulation of anti‐aging genes further supports a tissue‐induced aging program in the skin. Additionally, decreased triglyceride levels, key components of subcutaneous fat [35], are consistent with lipodystrophy in PL patients [36], while reduced unsaturated fatty acids may contribute to membrane rigidity and accelerated cellular aging [37].

Based on the pronounced metabolic alterations observed across multiple organs, we next examined whether these molecular signatures are reflected in the circulating metabolome, thereby enabling the identification of systemic, non‐invasive biomarkers relevant to PL pathology. Serum metabolomic analysis revealed significantly elevated levels of LPC species (such as LPC (18:1e) and LPC (18:2e)) and LysoPA in both PL patients and mouse models. These changes resemble aging‐ and senescence‐related features observed in long‐lived individuals. Specifically, the increase in LPC (16:0) and LPC (18:2), lipids typically found to be reduced in centenarians, supports the hypothesis that LPC accumulation is associated with features of premature aging [38, 39]. However, LPC (18:1e), which is not previously reported, showed consistent elevation in both PL and physiologically aged cohorts, indicating its potential as a robust aging‐associated biomarker. Moreover, LPC serves as a precursor to LysoPA, a bioactive lipid closely associated with fibrosis, inflammation, and thrombosis, further implicating its involvement in the systemic pathological progression of PL.

In an independent human cohort, we validated the upregulation of LPC and LysoPA as well as the reduction of D‐glucose 1‐phosphate. Moreover, NAD metabolism was significantly altered with reduced NAD and accumulation of its degradation products NAM and 2‐PY. NAD reduction has been demonstrated to impair SIRT1‐mediated deacetylation of p53 and other senescence regulators, potentially facilitating p16/p21‐dependent cell cycle arrest [40]. These metabolic profiles showed directional consistency with patterns observed in a cross‐sectional cohort of healthy elderly individuals.

In addition to the validated circulating biomarkers, we observed alterations in other metabolites previously linked to aging processes. For instance, taurocholate (TCA), a taurine‐conjugated bile acid, was consistently downregulated across all three organs in HGPS mice. Taurine, a conditionally essential amino sulfonic acid, is known for its antioxidative, anti‐inflammatory, and mitochondrial‐stabilizing functions [41, 42, 43], and disruption of the taurine–TCA–TUDCA axis may compromise tissue resilience and contribute to multi‐organ vulnerability in PL. Notably, the marked downregulation of TUDCA in the skin might partially explain the severe cutaneous dysregulation observed in our study. Since TUDCA acts as a potent cytoprotectant that maintains protein and lipid homeostasis, its depletion may exacerbate the accumulation of ceramides and the loss of subcutaneous triglycerides, leading to the compromised skin barrier and lipodystrophy characteristic of PL patients. This finding aligns with prior reports that bile acid supplementation (e.g., TUDCA) extends lifespan in progeroid models [44]. While taurine's role as a universal aging biomarker remains debated due to individual variability [45], our results suggest its protective effects may depend on maintaining this metabolic pathway, offering a potential therapeutic target. However, these observations are primarily based on omics‐level analyses and histological characterization, and further functional and mechanistic validation will be required to establish causal relationships.

Several limitations of this study merit acknowledgment. First, the tissue‐level metabolomic profiling was conducted in a relatively small cohort of HGPS mice, which constrains statistical power and warrants replication in larger cohorts. Second, all metabolic alterations reported here are associative in nature; the absence of functional intervention experiments (e.g., TUDCA supplementation, LPC/LysoPA pathway inhibition) means that causal roles for the identified pathways cannot be established from this dataset alone. Third, the study design is cross‐sectional, and we cannot infer longitudinal dynamics of metabolic change during disease progression. Finally, the causal relationship between the identified metabolic alterations — in particular, NAD depletion and LysoPA elevation — and cellular senescence remains to be experimentally established. Future longitudinal, multi‐strata, and interventional studies will be essential to fully establish the clinical utility of the biomarkers identified here.

## 4 Conclusion

This study provides a comprehensive multi‐organ and multi‐cohort metabolic characterization of progeroid laminopathy, revealing both tissue‐intrinsic alterations and systemic circulating biomarkers. Elevated LPC(18:1e) and LysoPA, together with reduced D‐glucose 1‐phosphate and NAD‐related metabolites, emerge as conserved markers of accelerated and physiological aging. Disruption of the taurine–TCA–TUDCA axis potentially further highlights metabolic vulnerability across organs. Collectively, these findings enhance the mechanistic understanding of progeroid syndromes and nominate actionable biomarkers and metabolic pathways with potential translational relevance to aging‐associated diseases.

## 5 Experimental Section

### 5.1 Animal Experiments and Organ Collection

All experiments were conducted following protocols approved by The Animal Ethics Committee of Zhejiang University (approval No. ZJU20230943). WT C57BL/6 mice (8 weeks) were purchased from Beijing Vital River Laboratory Animal Technology Co. Ltd. (Beijing, China). F0 heterozygous female and male C57BL/6‐Tg (*Lmna* ^*G608G*^) HClns/J mice were purchased from the Jackson Laboratory (Hemizygous for Tg (*Lmna* ^*G608G*^) HClns, 010667). Homozygous mice were obtained by mating homozygous males with heterozygous females. We selected F2 homozygous male mice (*Lmna* ^*G608G*^) for subsequent experiments (*n* = 3–5). Heart, Lung, skin and serum from 6‐month‐old male mice were used in this study. Each experiment was independently repeated at least three times.

### 5.2 Tissue Paraffin Histology

The heart, lung and skin were fixed in 4% paraformaldehyde (PFA) for 24 h, followed by dehydration using graded alcohols and embedding in paraffin. Cross‐sections with a thickness of 4–5 μm were prepared and mounted on charged slides for visualization using hematoxylin and eosin (H&E) staining (Solarbio, China). Additional staining was performed using Masson's trichrome (Solarbio, China). Images were acquired using the Aperio Image Scope 12.4.6 imaging system at magnifications of 5X and 20X and analyzed using Image software. Subsequently, further processing of the images was performed using the Adobe Illustrator 2022 software to achieve high‐quality and clear visuals.

### 5.3 Collection of Serum From PL Patients

This research was performed in compliance with the ethical standards set by the Children's Hospital at Zhejiang University School of Medicine and was approved by the Ethics Committee (Ethical Protocol Number: 2023‐IRB0186‐P‐01). The study strictly adhered to the principles delineated in the Declaration of Helsinki, and the reporting of the research followed the comprehensive EQUATOR guidelines (Simera et al. 2010). PL Patients were enrolled according to strict pre‐defined inclusion criteria. The most important inclusion criteria were as follows: age ≥ 1 year and classical or non‐classical HGPS through genetic testing (*LMNA* or *ZMPSTE24* mutation). Patients carrying the classical *LMNA* ^*G608G*^ were defined as classical HGPS, while other genes involved in nuclear envelope stability, such as *LMNA* (non‐G608G variants) or *ZMPSTE24* (Zinc Metallopeptidase STE24), leading to a progeroid phenotype defined as HGPS‐like PL. Serum from 32 participants was used for metabolomic analysis, with 14 individuals diagnosed with PL and 18 normal people. Serum from 52 participants was used for independent validation, with 26 individuals diagnosed with PL and 26 normal people.

### 5.4 Collection of Serum From Young and Old People

This research was performed in compliance with the ethical standards set by the Zhejiang Hospital and was approved by the Ethics Committee (Ethical Protocol Number: 2022‐IRB155K). Young (< 18 years old) and old (> 65 years old) adults, including male individuals (*n* = 6 young, *n* = 6 older) and female individuals (*n* = 10 young, *n* = 8 older) were recruited. All participants were physically healthy with no major medical illnesses or chronic medical illnesses. Serum samples were collected from these participants.

### 5.5 Total RNA Isolation and RT‒PCR

Total RNA was isolated from tissues using TRIzol reagent (Thermo Fisher Scientific, USA) and subsequently purified with chloroform following the manufacturer's guidelines. RNA concentrations were determined using a NanoDrop 1000 spectrophotometer (Thermo Fisher Scientific, USA). For cDNA synthesis, 0.5 μg of total RNA was reverse transcribed, and quantitative real‐time PCR was performed using SYBR Green Master Mix (Vazyme, China). mRNA expression levels were normalized to *β*‐actin as an internal control.

### 5.6 Untargeted Metabolomic Analysis

Untargeted metabolomic analysis of samples in each group was conducted using an ultra‐performance liquid chromatography quadrupole time‐of‐flight mass spectrometer (UPLC‐QTOF‐MS). Each sample (60 mg) was homogenized with 0.8 mL of methanol: water (4:1, V/V) containing 0.1% formic acid. Subsequently, 180 L of chloroform was added to the tissue homogenate, followed by 5 min of centrifugation at 13,000 g and 15 min of centrifugation to collect the supernatant, which was then concentrated using a vacuum concentrator. The samples were then dissolved in 0.7 mL of methanol: water (1:1, V/V). For quality control (QC) purposes, 100 μL of each sample was extracted to create QC samples. QC samples were added to every fifth sample to assess the stability of the UPLC‐QTOF‐MS system. (OPLS‐DA) was used to reduce data dimensionality. Metabolites with Variable Importance in Projection (VIP) values greater than 1 were selected as key features for subsequent analysis. The MetaboAnalyst platform was utilized for automatic normalization of metabolite data. The *p* < 0.05 and logFC>0.5 were considered as differentially expressed metabolites (DEMs). DEMs were then used to perform pathway enrichment using the KEGG database. Lipid‐class identification has been based on accurate mass, fragmentation analysis, relative retention times and ion mobility compared to the analysis of relevant standards. HyperGTest was used to evaluate whether differentially expressed lipids were enriched in various lipid classes.

### 5.7 Qualification of Metabolites Determined by UHPLC‐MS/MS

The dried metabolized extract was reconstituted in 100 μL 70% acetonitrile solution. An ultra‐performance liquid chromatography‐tandem mass spectrometry (UHPLC—MS/MS) method was developed for differential metabolite analysis using a UHPLC‐MS/MS system with the SCIEX QTRAP 6500 triple quadrupole mass spectrometer (SCIEX, USA) coupled to a Nexera X2 LC‐30AD detector (Shimadzu Corporation, Japan). This system utilized an Electrospray ionization (ESI) interface and a cooling autosampler. Operating conditions for the ESI were set as follows: temperature at 300°C, curtain gas at 30 psi, nebulizer gas, ion spray voltage (IS) at 5500 V/−4500 V, and spray gas and auxiliary heating gas at 35 psi, respectively. Each analyte was performed in multi‐reaction monitoring (MRM) parameters to determine the optimal conditions by fluidizing a single reference substance into an ESI source in either positive or negative ion mode. A gradient (0.5, 1.0, 2.0, 5.0, 10.0, 20.0, 50.0, 100, 500, 1000 ng/mL) was applied simultaneously to the temperatures of vaporizing. Sensitivity and specificity were optimized, and parent and daughter ions specific to each molecule, capillary voltages, and collision energies. Peak areas were used for calculations by Tracefinder 3.2 (Thermo Fisher Scientific, USA).

### 5.8 Qualification of NAD Metabolites Determined by UHPLC‐MS/MS

Dried metabolite extracts were dissolved in 100 μL of 70% acetonitrile. Metabolic profiling was conducted using a UHPLC‐MS/MS platform composed of a SCIEX QTRAP 6500 triple quadrupole mass spectrometer (SCIEX, USA) and a Nexera X2 LC‐30AD system (Shimadzu, Japan). The setup included an electrospray ionization (ESI) source and a temperature‐controlled autosampler. Detailed experimental procedures were performed as previously described in our previous studies [9].

### 5.9 Statistical Analysis

Statistical comparisons among the different groups were made using Student's t‐test. We performed normality tests (such as the Shapiro–Wilk test or Kolmogorov–Smirnov test) on the data to assess whether the distributions followed a normal curve. Based on the results of these tests, we determined that parametric tests were appropriate for our data. *p* < 0.05 were considered statistically significant.

## Author Contributions

L.H., N.S., and J.M. conceived and supervised the study. J.W., G.W., and L.G. collected the heart, lung, and skin tissue of HGPS and WT mice. X.L., Z.F., J.Y., and Z.P. performed the metabolome analyzes. X.Z. and Y.Z. conducted data analysis. X.Z., Y.Z., L.H., and N.S. co‐wrote the manuscript. All authors read and approved the final manuscript.

## Ethics Statement

All animal experiments were conducted in accordance with the guidelines of the Animal Ethics Committee of Zhejiang University (approval No. ZJU20230943). For human studies, the collection and use of patient serum samples were approved by the Ethics Committee of the Children's Hospital, Zhejiang University School of Medicine (Ethical Protocol No. 2023‐IRB0186‐P‐01). The collection of serum samples from young and elderly participants was approved by the Ethics Committee of Zhejiang Hospital (Ethical Protocol No. 2022‐IRB155K). All procedures adhered to the principles of the Declaration of Helsinki.

## Conflicts of Interest

The authors declare no conflicts of interest.

## Supporting information


Supporting Information S1

**Table S1:** Metabolomic Profiling Reveals Lipid and Metabolic Dysregulation in HGPS Mice, related to Figure 3.

**Table S2:** Serum Metabolome Profiling Reveals Systemic Metabolic Dysregulation in PL, related to Figure 4.

## Data Availability

The data that support the findings of this study are openly available in HGPS and WT mouse heart, lung and skin Lipidome data at https://ngdc.cncb.ac.cn/omix/preview/0xgL4yss, reference number OMIX010373.
