# Supplementary material for: Metabolic Profiling Reveals Organ‐Specific Molecular Pathologies and Aging‐Associated Biomarkers in Progeroid Laminopathy
Source: Smart Med. 2026 Jul 11;5(3):e70042. doi: 10.1002/smmd.70042 (PMC13387292; doi:10.1002/smmd.70042)
Supplement: Supplementary file 1 — Supporting Information S1 [file SMMD-5-e70042-s002.docx]

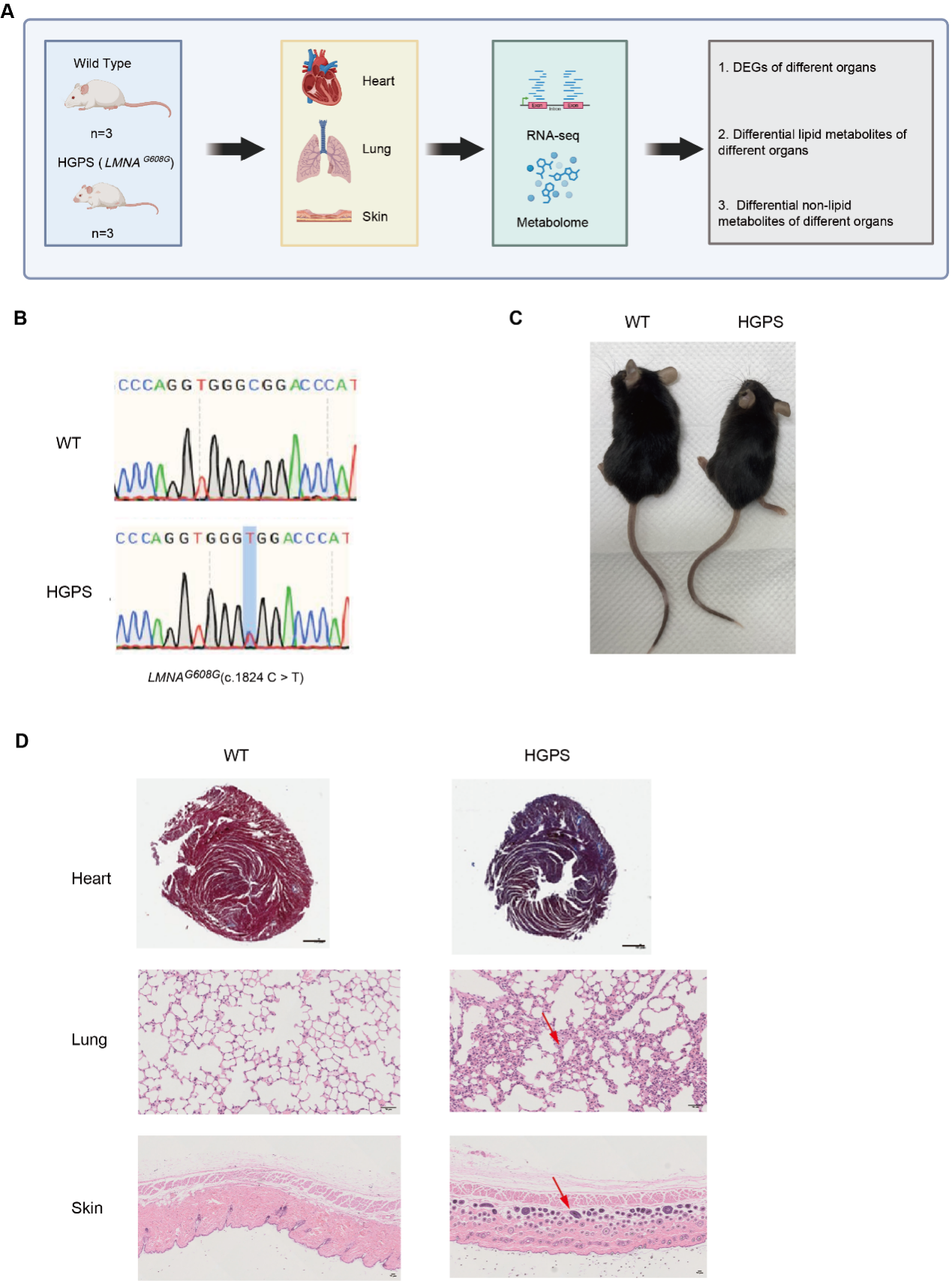


**Fig.S1 Phenotypic Characterization of HGPS Mice.** (A) Technical roadmap for transcriptomic and metabolomic profiling of heart, lung, and skin in HGPS mice. (B) DNA sequencing results of WT mice and HGPS mice around LMNA mutated regions. (C) Comparative analysis of morphological features between WT and HGPS mice. (D) Masson staining of heart and H&E staining of lung and skin of WT and HGPS mice, scale =200 μm.

­­


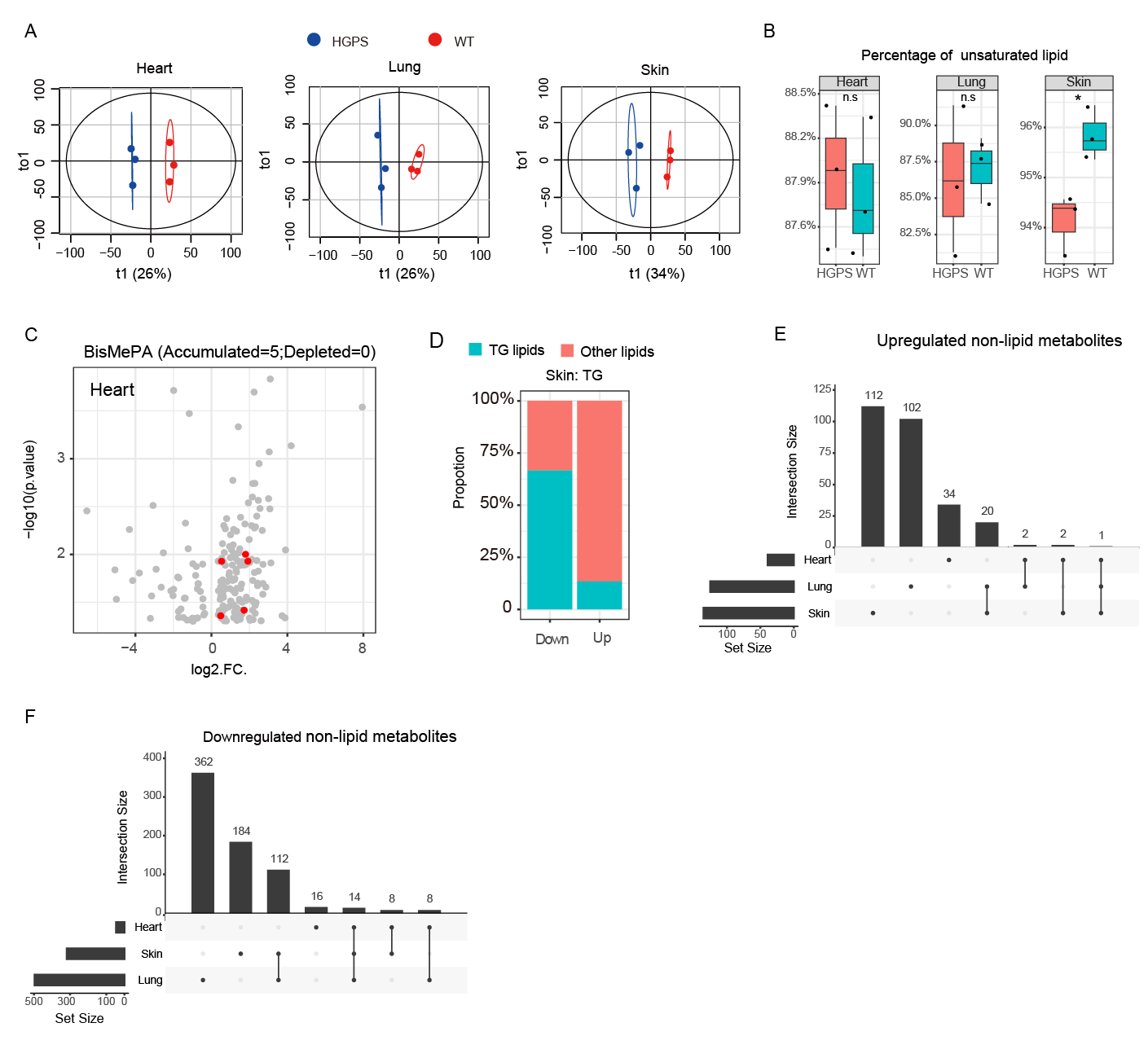


**Fig. S2 Lipidomic and metabolomic profiling reveals distinct organ-specific alterations in HGPS mice.** (A) OPLS-DA analysis of HGPS and WT samples across different organs. (B) Proportion of unsaturated fatty acid content in different organs. (C) Volcano plot of differential lipids in the heart (HGPS vs WT), with red indicating differential lipids belonging to the BisMePA class and grey indicating those not belonging to the BisMePA class. (D) Percentage of TG-class differential lipids among all upregulated or downregulated differential lipids in skin tissue. (E) Upset plot showing the number of significantly upregulated hydrophilic metabolites unique to or shared across different organs. (F) Upset plot showing the number of significantly downregulated hydrophilic metabolites unique to or shared across different organs.


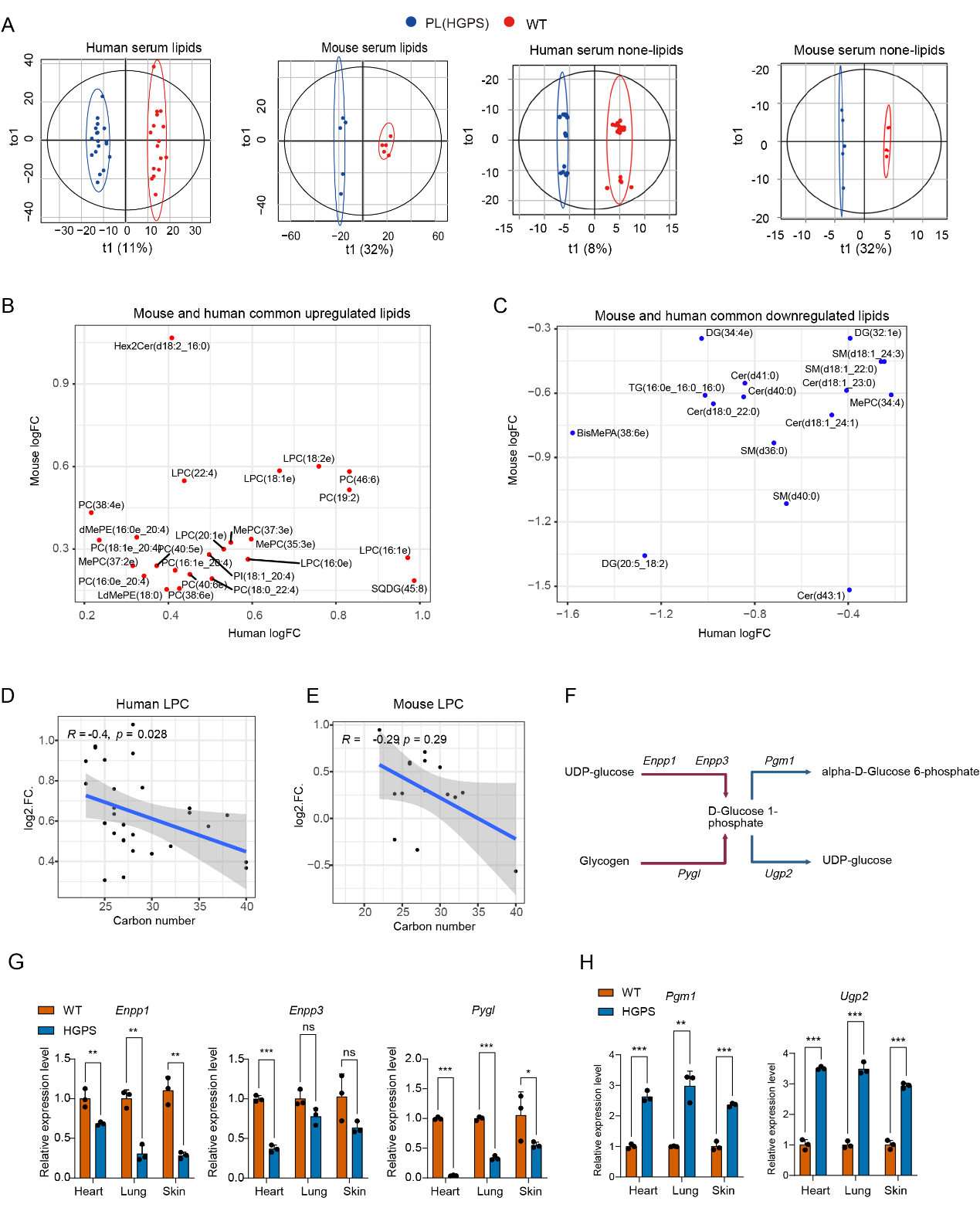


**Fig. S3 Circulating lipid and glucose metabolic alterations in PL patients and mouse models reveal conserved aging signatures.** (A) OPLS-DA analysis of serum samples from humans and mice, with red indicating WT and blue indicating PL (including HGPS) serum samples. (B) Common upregulated lipids in serum samples from PL patients and HGPS mice, and the degree of change in these lipids in both human and mouse serum. The X-axis represents the fold change of differential lipids in the serum of PL patients compared to normal controls, while the Y-axis represents the fold change of differential lipids in the serum of HGPS mice compared to WT mice. (C) Common downregulated lipids in serum samples from PL patients and mice, and the degree of change in these lipids in both human and mouse serum. The X-axis represents the fold change of differential lipids in the serum of PL patients compared to normal controls, while the Y-axis represents the fold change of differential lipids in the serum of HGPS mice compared to WT mice. (D) Correlation between differential expressions of LPCs and the number of carbon atoms in their molecular structure in PL patients' serum. (E) Correlation between differential expressions of LPCs and the number of carbon atoms in their molecular structure in mouse HGPS serum. (F) Metabolic pathway of D-glucose 1-phosphate, with associated enzymes. Burgundy and blue arrows indicate enzymes involved in its synthesis and conversion, respectively. (G) Relative expression levels of *Enpp1*, *Enpp3*, and *Pygl* in heart, lung, and skin organs of WT (orange) and HGPS (blue) mice. (H) Relative expression levels of *Pgm1* and *Ugp2* in heart, lung, and skin organs of WT (orange) and HGPS (blue) mice. ns: no significant. **: p<0.05. **: p<0.01. ***: p<0.001.*


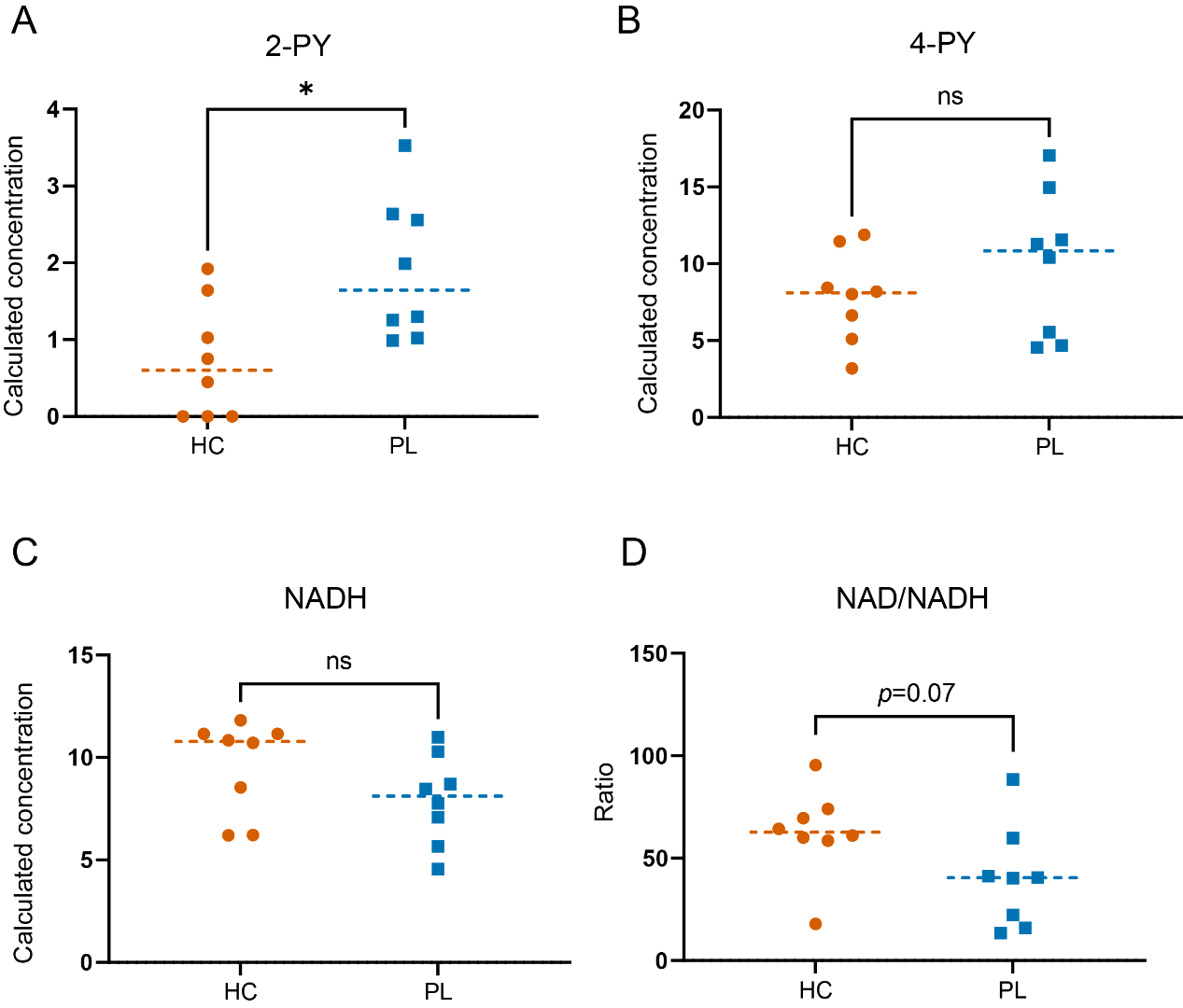


**Fig. S4** Quantification of NAD-related metabolites in serum from HC, orange) and PL patients (blue). (A) Calculated concentration of 2-pyridone (2-PY). (B) Calculated concentration of 4-pyridone (4-PY). (C) Calculated concentration of NADH. (D) Calculated the ratio of NAD/NADH.

ns: no significant. *: *p<0.05*.
